# Neutrophil Migration into the Infected Uroepithelium Is Regulated by the Crosstalk between Resident and Helper Macrophages

**DOI:** 10.3390/pathogens5010015

**Published:** 2016-02-04

**Authors:** Kristina Zec, Julia Volke, Nirojah Vijitha, Stephanie Thiebes, Matthias Gunzer, Christian Kurts, Daniel Robert Engel

**Affiliations:** 1 Institute of Experimental Immunology and Imaging, Department Immunodynamics, University Hospital Essen, University Duisburg-Essen, Essen 45141, Germany; kristina.zec@uk-essen.de (K.Z.); julia.volke@stud.uni-due.de (J.V.); nirojah.vijitha@uk-essen.de (N.V.); thiebes@immunodynamics.de (S.T.); Matthias.Gunzer@uni-due.de (M.G.); 2 Institute of Experimental Immunology, University Hospital Bonn, Bonn 53127, Germany; ckurts@uni-bonn.de

**Keywords:** crosstalk, transepithelial migration, Ly6C^+^ and Ly6C^−^ macrophages, neutrophils, uropathogenic *E. coli* (UPEC)

## Abstract

The antibacterial defense against infections depends on the cooperation between distinct phagocytes of the innate immune system, namely macrophages and neutrophils. However, the mechanisms driving this cooperation are incompletely understood. In this study we describe the crosstalk between Ly6C^+^ and Ly6C^−^ macrophage-subtypes and neutrophils in the context of urinary tract infection (UTI) with uropathogenic *E. coli* (UPEC). Ly6C^−^ macrophages acted as tissue resident sentinels and attracted circulating phagocytes by chemokines. Ly6C^+^ macrophages produced tumor necrosis factor (TNF) that licensed Ly6C^−^ macrophages to release preformed CXCL2, which in turn caused matrix metalloproteinases (MMP-9) secretion by neutrophils to enable transepithelial migration.

## 1. Introduction

The phagocytes of the innate immune system, macrophages and neutrophils, contribute to antibacterial defense, but their functional specialization and cooperation is unclear. Neutrophils are bone marrow–derived, circulate through the blood, and enter infected tissues by penetrating the vascular endothelium, which has been studied extensively [1,2,3,4,5,6], but the mechanisms guiding neutrophils within tissues toward microbes are less well known. Of particular importance is neutrophil migration into tissue epithelia, a barrier to the outside world and a preferential entry site for the bacteria, which requires traversing the epithelial basement membrane and cleavage of collagen IV [1,7,8]. Besides matrix metalloproteinases (MMPs), chemokines are involved in crossing these membranes [9,10]. Furthermore, *in vitro* studies suggest that some MMPs are regulated by chemokines [11,12,13] and *vice versa* [14]. However, the role of chemokines and MMPs in epithelial neutrophil migration *in vivo* is incompletely understood. Macrophages are an important source of chemokines in bacterial infections, such as CXCL1 and CXCL2 [15,16,17,18]. Hence, it was assumed that macrophages and neutrophils cooperate during infections [19,20], but a coherent model on how they communicate and share the anti-infectious tasks is presently lacking. Tissue-resident macrophages, partially originating from embryonic progenitors, are present in all tissues [21,22]. During infections, they receive a back-up from inflammatory monocytes released from bone marrow which further differentiate into macrophages once within tissues [23]. Inflammatory macrophages can be distinguished from tissue-resident macrophages by the marker Ly6C until it is down-regulated after one to two days [24]. Bacterial infections of the urinary tract (UTI) are among the most prevalent infections and affect more than 25% of the population in developed countries, especially young females [25]. They mainly result from uropathogenic *E. coli* (UPEC) ascending through the urethra into the bladder, where they first invade the uroepithelium [26]. UPEC can persist intracellularly and cause relapsing infections [27], which are associated with an increased risk for bladder cancer. Their ascension into the kidney causes pyelonephritis, which can progress to renal failure [28,29]. The defense against UTI depends on neutrophils [30]. However, macrophages produce proinflammatory cytokines, such as tumor necrosis factor (TNF), during bacterial infections [18,31,32,33], and the roles of macrophages and TNF in UTI have so far been unknown. We have studied the interplay of neutrophils and macrophages in a murine model of UTI of the bladder and discovered that tissue-resident Ly6C^−^ and pro-inflammatory Ly6C^+^ macrophages play distinct roles in antibacterial immunity. Their TNF-mediated crosstalk regulates neutrophil migration into the infected uroepithelium and facilitates optimal antibacterial defense. We propose a model of tri-cellular crosstalk in innate cellular immunity [34].

## 2. Results and Discussion

**Figure 1:**
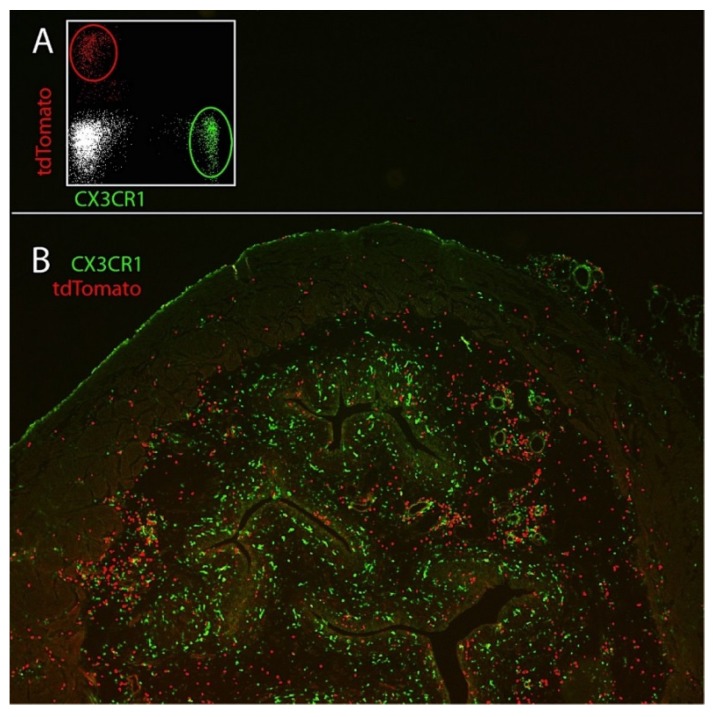
Ly6g ^tdTomato^ ^IVM^ Cx3cr1 ^GFP^ mice were transurethrally infected with UPECs. One day later, GFP-expressing macrophages and tdTomato-expressing neutrophils were analyzed by flow cytometry (**A**) and by microscopy (**B**). Analysis of GFP-expressing macrophages revealed that these cells are equally distributed underneath the epithelium. In contrast, aggregates of tdTomato-expressing neutrophils were found within the infected epithelium. We also observed clustering of neutrophils around endothelial structures, indicating ongoing recruitment from the circulation.

To study the roles of macrophages and neutrophils, we crossed *Cx_3_Cr1^GFP/+^* mice [35] with *Ly6g ^tdTomato IVM^* mice [36]. After transurethral instillation of UPECs into these mice, we found CX_3_CR1-expressing macrophages and also tdTomato^+^/Ly6G^+^ neutrophils by flow cytometry and by microscopy (Figure 1A,B). Further analysis by flow cytometry revealed that bladders of uninfected mice already contained CX3CR1^+^ Ly6C^−^ macrophages whereas the infected bladders contained significant numbers of both CX3CR1^+^ Ly6C^+^ macrophages and Ly6C^+^ Ly6G^+^ neutrophils observable after 2 h and reaching maximum numbers after 24 h due to their rapid recruitment from the circulation, as demonstrated by the absence of *in situ* proliferation [34]. The numbers of Ly6C^−^ macrophages remained constant throughout the course of infection.

### 2.1. Neutrophils Internalize Uropathogenic E. coli

The importance of these phagocyte types for the uptake of UPEC was determined by using green fluorescent protein (GFP)-expressing bacteria. Almost 90% of the phagocytized UPECs were detected within neutrophils 6 h after infection. Therefore, we speculated that Ly6C^−^ or Ly6C^+^ macrophages might regulate the antibacterial defense by producing neutrophil-attracting chemokines and found that the Ly6C^−^ macrophages produced the neutrophil attractors MIF, CXCL1, CXCL2, and CXCL6 while Ly6C^+^ macrophages mainly produced TNF. To prove that the presence of the Ly6C marker truly discriminates between two distinct macrophage populations, we infected *Ccr2^−/−^* mice, which lack Ly6C^+^ macrophages [23], and mice treated with clodronate liposomes, which selectively deplete Ly6C^+^ macrophages with UPEC. In both cases, no Ly6C^+^ macrophages were detected in the bladder 6 h after infection, supporting the notion that Ly6C^−^ and Ly6C^+^ macrophages were functionally distinct macrophage types.

### 2.2. TNF-Dependent Neutrophil Transepithelial Migration

We next determined the roles of the chemokines produced by Ly6C^−^ macrophages by blocking them with neutralizing antibodies. CXCL1 and its receptor CXCR2 were critical for neutrophil recruitment. MIF contributed to such recruitment, whereas CXCL2 and CXCL6 were not required. Ly6C^−^ macrophages produce CCL2 as well, attracting Ly6C^+^ macrophages. Bacterial clearance of the bladder was impaired in the absence of neutrophils (αCXCL1, αMIF, and *Cxcr2^−⁄−^* mice), as well as in mice lacking or depleted of Ly6C^+^ macrophages. In summary, Ly6C^−^ macrophages produced the chemokines that attracted neutrophils and Ly6C^+^ macrophages into the infected bladder, and both recruited phagocyte types were important for antibacterial defense. To investigate whether the importance of Ly6C^+^ macrophages is mediated in their main product TNF, we infected *Tnfr^−⁄−^* mice deficient for both TNF receptors and found that the bacterial load in their bladders was much higher than in wild-type controls and that the number of infected uroepithelial cells, indicative of the persistence of UTI, was increased both in *Tnfr^−⁄−^* mice and in the absence of the TNF-producing Ly6C^+^ macrophages. Histological analysis further revealed that in *Tnfr^−⁄−^* mice and in mice depleted of TNF-producing Ly6C^+^ macrophages, neutrophils were unable to enter and transmigrate through the uroepithelium into the bladder lumen. Reconstitution of infected *Tnfr^−⁄−^* mice either with recombinant TNF or Ly6C^+^ macrophages restored the neutrophil entry into the uroepithelium.

### 2.3. Ly6C^−^ Macrophages Produce CXCL2

To determine whether TNF acts directly on neutrophils and subsequently permits uroepithelial transmigration, TNFR-competent and -deficient neutrophils were adoptively transferred into infected wild-type mice. However, both of these neutrophils were able to enter the bladder lumen, demonstrating the dispensability of the TNFR on neutrophils. Generation of bone marrow chimeras in which hematopoietic and/or non-hematopoietic cells lacked TNFR and their subsequent infection showed that transepithelial neutrophil migration required TNFR expression expressed by bone marrow–derived cells. The analysis of expression of molecules affecting neutrophil migration in wild-type or *Tnfr^−⁄−^* mice showed that CXCL2 was markedly reduced in *Tnfr^−⁄−^* mice, indicating that TNF induced CXCL2. The cellular source of CXCL2 according to our flow cytometric analysis was Ly6C^−^ macrophages. Immunofluorescence microscopy also showed that only Ly6C^−^ macrophages, but not epithelium, produced CXCL2 not only in infected but also in uninfected animals, indicating the existence of preformed intracellular pools. Thus, TNF acts on Ly6C^−^ macrophages to induce CXCL2. Furthermore, transurethral inoculation of CXCL2 into the bladder of infected *Tnf^−⁄−^* mice restored neutrophil migratory ability, demonstrating that CXCL2 act downstream of TNF.

### 2.4. Neutrophil Migration across Epithelium Depends on MMP-9

Next, we investigated the mechanisms underlying how neutrophils migrate across the epithelial basement membrane. These basement membranes are comprised mainly of collagen IV, which is degraded by metallomatrix proteinase 9 (MMP-9) [14], and CXCR2 ligands have been shown to induce MMP-9. Indeed, histological sections of infected *Mmp-9^−⁄−^* mice showed that neutrophils accumulated underneath the uroepithelium and failed to enter it. Neutrophil-intrinsic MMP-9 was necessary and sufficient for epithelial migration, demonstrated by transferring MMP-9–competent neutrophils into infected *Mmp-9^−⁄−^* mice, and MMP-9–deficient neutrophils into infected MMP-9–competent mice. Finally, neutrophils were stimulated with CXCL2 *in vitro* which caused MMP-9 secretion in a concentration-dependent manner, demonstrating that CXCL2 permits epithelial neutrophil migration by the induction of MMP-9.

### 2.5. Three-Cellular Crosstalk between Ly6C^−^ and Ly6C^+^ Macrophages with Neutrophils

Taken together, our findings documented the following sequence of events during the first 4 h of the phagocyte response against UTI: (1) resident Ly6C^−^ macrophages sensed the infection and produced chemokines that recruited neutrophils and Ly6C^+^ macrophages; (2) recruited Ly6C^+^ macrophages produced TNF in response to the infection; (3) TNF induced the release of preformed CXCL2 by Ly6C^−^ macrophages; (4) CXCL2 caused MMP-9 secretion by neutrophils, which allowed these cells to cross the epithelial basement membrane in order to combat the infection [34]. The chemokines that bind CXCR2 are often considered redundant. We found that Ly6C^−^ macrophages produced several such chemokines, which played distinct chemotactic roles in regulating neutrophil migration in UTI: CXCL1, and to a lower extent MIF, caused endothelial migration, whereas CXCL2 by Ly6C^−^ macrophages played a non-redundant role in epithelial migration. The ability of CXCL2 to induce the secretion of MMP-9 [12] in the immediate vicinity of the epithelial basement membrane explains how neutrophils can penetrate collagen IV–rich epithelial basement barriers. Our findings identify CXCL2 and MMP-9 as TNF-regulated gatekeepers for epithelial neutrophil migration. It remains to be seen whether CXCL2 induction is important also in other TNF-dependent bacterial infections, for example listeriosis [37] or tuberculosis [38].

## 3. Experimental Section

### 3.1. Mice

Female *Cx_3_Cr1^GFP/+^* [35] mice were crossed to *Ly6g ^tdTomato^^IVM^* [36] mice in specific pathogen-free condition. Eight-week-old mice were used for the experiments in accordance with local governmental review boards (Bezirksregierung Köln, Landesamt für Natur, Umwelt und Verbraucherschutz NRW in Recklinghausen, Germany).

### 3.2. UPEC Model

The uropathogenic *E. coli* (UPEC) strain 536 (O6:K15:H31) originating from a patient suffering from urinary tract infection was used for induction of bladder infection. The performed the murine urinary tract infection as described previously [34].

### 3.3. Histology

Immunofluorescence microscopy in cryosections: we embedded fixed bladders of *Cx_3_Cr1^GFP/+^ Ly6g ^tdTomato IVM^* mice into Tissue-Tec (Sakura). Frozen blocks were cut into 5 mm sections and mounted on poly-L-lysin-coated glass slides (Menzel). We analyzed the sections using the Axio Observer.Z1/Apotome (Zeiss).

## 4. Conclusions

These results demonstrated that the antibacterial neutrophil response is coordinated by two macrophage subsets with distinct tasks: the Ly6C^−^ macrophages acted as tissue-resident sentinels and attracted circulating phagocytes by chemokines. The Ly6C^+^ macrophages did not directly participate in bacterial elimination but instead licensed the Ly6C^−^ macrophages to send neutrophils into the frontline of infection, the uroepithelium. These findings are of major importance for understanding the coordination of an innate immune response in UTI, but also in other inflammatory settings.
